# Examining Associations between Adverse Childhood Experiences and Posttraumatic Stress Disorder Symptoms among Young Survivors of Urban Violence

**DOI:** 10.1007/s11524-022-00628-4

**Published:** 2022-06-14

**Authors:** Loni Philip Tabb, John A. Rich, Daria Waite, Cinthya Alberto, Erica Harris, James Gardner, Nina Gentile, Theodore J. Corbin

**Affiliations:** 1 Department of Epidemiology & Biostatistics, Dornsife School of Public Health, Drexel University, Philadelphia, PA USA; 2 Center for Nonviolence & Social Justice, Dornsife School of Public Health, Drexel University, Philadelphia, PA USA; 3 Department of Health Management & Policy, Dornsife School of Public Health, Drexel University, Philadelphia, PA USA; 4 Department of Emergency Medicine, Albert Einstein Medical Center, Philadelphia, PA USA; 5 Department of Emergency Medicine, MedStar Washington Hospital Center, Washington, DC USA; 6 Department of Emergency Medicine, Temple University Lewis Katz School of Medicine, Philadelphia, PA USA; 7 Department of Emergency Medicine, Rush University Medical Center, Chicago, IL 60612 USA

**Keywords:** Adverse childhood experience (ACE), Posttraumatic stress, Chronic trauma, Posttraumatic stress disorder (PTSD), Youth, African American, Trauma-informed intervention

## Abstract

Our study examines the association between Adverse Childhood Experience (ACE) exposure and posttraumatic stress disorder (PTSD) symptoms among survivors of violence. In this cross-sectional study, an ACE questionnaire and PTSD Checklist for DSM-5 (PCL-5) were completed by 147 participants ≤ 3 months after presenting to a Philadelphia, PA emergency department between 2014 and 2019 with a violent injury. This study treated ACEs, both separate and cumulative, as exposures and PTSD symptom severity as the outcome. Most participants (63.3%) met criteria for provisional PTSD, 90% reported experiencing ≥ 1 ACE, and 39% reported experiencing ≥ 6 ACEs. Specific ACEs were associated with increasing PCL-5 scores and increased risk for provisional PTSD. Additionally, as participants’ cumulative ACE scores increased, their PCL-5 scores worsened (*b* = 0.16; *p* < 0.05), and incremental ACE score increases predicted increased odds for a positive provisional PTSD screen. Results provide further evidence that ACEs exacerbate the development of PTSD in young survivors of violence. Future research should explore targeted interventions to treat PTSD among survivors of interpersonal violence.

**Supplementary Information:**

The online version contains supplementary material available at 10.1007/s11524-022-00628-4.

## Background

Each year in the United States (US), approximately 1.5 million individuals present to hospitals nationwide for treatment of nonfatal assault injuries [1]. Rates of violence in the US vary by location where we observe higher homicide rates in urban areas compared to suburban or rural areas [2]. Over 80% of the 13,958 firearm homicides in the US in 2018 occurred in urban areas, and disparities are larger in certain areas of large cities (e.g., racially and ethnically diverse neighborhoods) [1]. Violence is associated with socioeconomic risk factors that disproportionately impact racial and ethnic groups in the US [3]. Through poverty and state sanctioned residential segregation, African Americans reside in some of the poorest neighborhoods in urban areas throughout the US with high rates of homicide [4].

African American males and youth in the US disproportionately carry the largest burden of violence exposure. Black youth and young adults ages 10-24 made up about 3.2% of the US population in 2020, however, they made up 9.2% of all hospital patients presenting for treatment of a nonfatal assault-related injury and 17.2% of all homicide victims over the past decade [1, 5]. Contributing factors to this disproportionate burden include historical, present day, and persistent structural racism (e.g., racial segregation), social inequity, and poverty [6]. For instance, in Philadelphia’s safest police district, which is approximately 85% White, there were no reported firearm homicides, whereas in the most violent district, where 90% of residents identify as African American, there were over 180 shooting victims and 40 firearm deaths [7].

Adverse Childhood Experiences (ACEs) are traumatic events that occur during childhood (0–17 years) and include experiencing violence, abuse, or neglect in the home and/or in the community [8]. ACEs are associated with poor health outcomes, including chronic health conditions in adulthood [9]. Additionally, studies have found a relationship between ACE exposure and mental/behavioral health outcomes such as alcohol and drug use, depression and/or anxiety symptoms, poor sleep quality, psychological distress, and poor self-rated health [9–12], ACEs also increase the probability of lifetime posttraumatic stress disorder (PTSD) and symptoms, particularly among racial, ethnic minority, and low-income populations in the US [13, 14]. Overall, ACEs contribute negatively to mental health, health outcomes, and quality of life. Assault survivors are at higher risk of experiencing PTSD symptoms after a subsequent traumatic event [15–17]. Violence exposure is also associated with poor psychological health, including depression and/or anxiety, and aggression [18].

PTSD is a mental health condition that can develop in individuals who have experienced or been exposed to a traumatic event, including ACEs or a violent injury [19, 20]. Higher rates of interpersonal violence among urban residents increases their risk of developing PTSD symptoms [18]. Furthermore, studies have shown that being traumatized and/or having PTSD is a risk factor for future victimization or involvement in violence [21–24]. Survivors of violence who are traumatized may cope with trauma symptoms through self-medication with substances and/or with weapon carrying for self-protection [18]. PTSD can also exert an intergenerational impact if parental trauma leads to ACEs for younger generations. This cycle of PTSD and ACEs becomes challenging to break in areas with limited economic and social opportunities for historically oppressed communities.

To examine the association between ACEs and PTSD among young survivors of violence, we analyzed data from Healing Hurt People (HHP), a trauma informed HVIP [25], to examine if ACEs were associated with PTSD in Philadelphia, PA. This cross-sectional study aimed to assess the prevalence of ACEs among young violently injured participants, determine associations between ACE exposure and posttraumatic stress symptoms after violent injury, and inform the practice and future direction of HVIPs, emergency medicine, primary care, and injury prevention research and policy.

## Methods

### Study Design and Setting

This was a cross-sectional study. Participants were recruited from three level 1 trauma centers in Philadelphia, PA. Philadelphia’s 2018 homicide rate was high compared to the national rate (22.28/100,000 vs 5.76/100,000, respectively) and other counties containing large US cities [1, 26–28]. Though Philadelphia is racially and ethnically diverse (42.3% non-Hispanic Black, 34.6% non-Hispanic White, 14.5% Hispanic, 7.2% Asian), the homicide mortality rate among African Americans is nearly tenfold that among non-Hispanic White people and double the rate among Hispanics [27, 28]. Pennsylvania’s 2016 Behavioral Risk Factor Surveillance System findings reveal that more non-Hispanic Black people had at least one ACE compared to non-Hispanic White people (58.6% vs. 45.1%, respectively) [28, 29]. Two of the current study’s trauma centers, Temple University Hospital (Temple) and Einstein Medical Center (Einstein), are located in North Philadelphia, an area with the city’s highest emergency department encounter rates [30]. The population within these centers’ ZIP codes is predominately Black or African American (Einstein 85.8% and Temple 59.2%), with approximately one-third of the population between ages 15 and 34 years (Einstein 32% and Temple 29.2%) [31, 32]. The third hospital, Hahnemann University Hospital (Hahnemann), which closed in 2019 post-data collection, was located in Center City Philadelphia. While Hahnemann’s ZIP code demographics differed from the other sites (i.e., less than 5% Black or African American and over 50% ages 15-34 years) [33], the center was accessible due to its proximity to several public transportation transit lines. Hahnemann was also considered one of Philadelphia’s safety-net hospitals, providing care to individuals from underserved communities [34].

### Participants

The study population consisted of 147 survivors of intentional injury between the ages of 18 and 33, recruited from Philadelphia level 1 trauma centers between 2014 and 2019. Intentional injury was defined as a gunshot wound, stab wound, or blunt force trauma inflicted by another person, based on the survivor’s self-report during presentation to the hospital. Cases of intimate partner violence or sexual assault, and patients exhibiting psychosis were excluded from the present study, as were patients who were actively suicidal or homicidal, not medically stable enough to participate, intoxicated during recruitment attempts, or in police custody/incarcerated during the study enrollment window.

If seen in the emergency department or the inpatient trauma unit, participants were screened for eligibility by research staff and recruited at bedside after being medically cleared and/or stabilized. Patients discharged before staff could proceed with in-person recruitment were identified through electronic medical records and contacted via phone by a trained research staff member or social worker. Participants were enrolled and initial data was collected within three months of their presenting injury. Clinical scales were administered using an audio computer assisted self-interviewing (ACASI) survey. The Institutional Review Board at each of the hospitals involved approved their respective protocols for the study.

### Variables

#### Sociodemographic Variables

Sociodemographic variables included gender, age, and site. Gender was dichotomized into categories “male” and “female.” Age was dichotomized into participants’ ages 18–24 years and those ages 25–33 years based on the study population’s age distribution. Site was operationalized as either the trauma center at which participants presented with a violent injury or the institution with which participants were connected post-injury. Sites were specified as Hahnemann University Hospital (Hahnemann), Temple University Hospital (Temple), and Einstein Medical Center (Einstein).

#### Self-Rated Health

Assessment of self-rated health was adopted from the Behavioral Risk Factor Surveillance System (BRFSS) [35] and measured using question “Would you say that in general your health is ____.” The responses were on an ordinal scale of “poor,” “fair,” “good,” “very good,” and “excellent.” For this analysis, we dichotomized self-rated health into good health (“good,” “very good,” and “excellent”) and poor health (“fair” and “poor”).

#### Adverse Childhood Experiences — Individual Assessment

To assess ACEs, we used a modified version of the CDC BRFSS ACE Module [35]. The CDC BRFSS ACE Module does not include items on neglect. To capture dimensions of neglect, we added the emotional neglect item from the CDC-Kaiser ACE Study survey [36]. The assessment captured adverse experiences prior to the age of 18. Ten ACEs were assessed covering nine categories, including emotional abuse, physical abuse, sexual abuse, mental illness in the household, incarcerated household member, substance abuse in the household (alcohol; drug use/abuse), parental separation or divorce, domestic violence, and emotional neglect.

Each individual ACE was captured if participants responded affirmatively. All questions related to ACEs are presented in the study’s supplemental material, accessible online (Table A1).

#### Adverse Childhood Experiences — Cumulative Assessment

Total ACE score for each participant was a sum of positive responses to individual ACE items with a possible score range of 0 (no ACEs affirmed) to 10 (all ACEs affirmed). Previous literature indicates that scores of 4 or greater are associated with an increased likelihood of chronic diseases in adulthood [36]. For our analysis, similar to Brockie et al. (2015), total ACE scores were categorized into three different exposure levels: low (0–2 ACEs), moderate (3–5 ACEs), and high (> 6 ACEs) [37].

#### Posttraumatic Stress Disorder Symptoms

PTSD symptoms were assessed using the PTSD Checklist for the DSM V (PCL-5), a 20-question measure based on DSM-5 criteria for PTSD, employing a 0–4 rating scale for each symptom and yielding a numeric total score of symptom severity [38]. This score was utilized to examine posttraumatic stress as a continuous variable. Furthermore, positive cases for provisional PTSD were defined as a total PCL-5 score of 33 or higher. As such, posttraumatic stress was also examined as a categorical variable, dichotomized as “positive provisional PTSD” (i.e., meeting clinical criteria for disorder) and “negative provisional PTSD.” PCL-5 total scores have demonstrated a high level of diagnostic utility for predicting a formal PTSD diagnosis compared to the Clinically Administered PTSD Scale (CAPS-5) clinical interview [39]. Overall, the instrument has been shown to be valid, reliable, and psychometrically sound [39, 40].

### Statistical Analysis

Descriptive statistics were used to characterize the study participants. Means and standard deviations were utilized for continuous measures, while counts and percentages were utilized for categorical measures.

#### Individual ACEs and Posttraumatic Stress

Separate logistic regression models were used to examine the relationship between individual ACEs and PTSD case status, where each model was fully adjusted for gender, age, site, and self-rated health. This resulted in ten models that examined the association between predictor variables (i.e., emotional abuse, physical abuse, sexual abuse, mental illness in the household, incarcerated household member, substance abuse in the household–alcohol, substance abuse in the household–drugs, parental separation or divorce, domestic violence, and emotional neglect) individually, and the outcome variable provisional PTSD case status. Odds ratios (OR) and 95% confidence intervals (CI) were utilized, with individual estimated associations graphically displayed, to further show compare the association between each ACE and PTSD case status. Additionally, we examined the association between each individual ACE and outcome variable PCL-5 score, measured continuously, using linear regression models in which estimated mean differences, standard errors, and *p*-values were reported to determine statistically significant relationships.

#### Cumulative ACEs and Posttraumatic Stress

To examine cumulative ACEs and the odds of positive provisional PTSD case status, we examined two logistic regression models. The first model focused on the predictive value of cumulative ACEs, as measured by total ACE score. The second model focused on the predictive value of low, moderate, or high ACE exposure categorization, with the low ACE exposure participants serving as the reference group. Both models, like the individual ACE logistic regression models, were fully adjusted for gender, age, site, and self-rated health. Finally, we examined the association between cumulative ACEs and total PCL-5 score using linear regression models.

#### Multicollinearity and Statistical Significance

The variance inflation factor (VIF) was used for all measures considered in each regression models to gauge potential multicollinearity [41]. All measures resulted in VIF values below 10 — indicative of little to no multicollinearity present in the models considered. Statistical significance was set at 0.05, and all analyses were conducted using R [42].

## Results

### Sample Characteristics

**Fig. 1:**
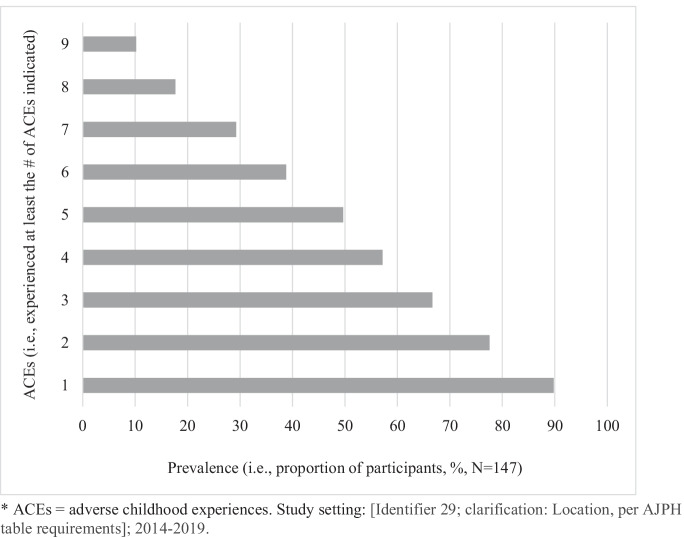
Distribution of ACEs (represented by experiencing at least the number of ACEs). *ACEs = adverse childhood experiences. Study setting: Philadelphia, PA; 2014-2019.

Study participants’ characteristics are presented in Table 1. Most participants were male (67.3%) and African American (73.5%), with an age distribution roughly split between those aged 18–24 years and those 25–33 years. Most participants enrolled from the Temple site (37.4%), while Hahnemann and Einstein sites enrolled 32.7% and 29.9%, respectively. Slightly over 70% of participants considered themselves in “good” health compared to 27.9% reporting “poor” health status. The average PCL-5 score was 37.1 (SD = 18.8), with 63.3% of participants meeting symptom criteria for provisional PTSD. Participants reported an average of 4.5 ACEs, with 38.8% having a high ACE categorization. Figure 1 displays the distribution of ACEs reported by study participants. Out of the 147 study participants, 90% (*n* = 132) experienced at least 1 ACE, and 10% (*n* = 15) experienced at least 9 ACEs. Over half of the participants experienced 4 or more ACEs.

**Table 1:** Baseline characteristics of study participants, *N* = 147

**Characteristic**	***n*** **(%)**	**Mean (SD)**
Gender		
Male	99 (67.3)	
Female	48 (32.7)	
Age (years)		
18–24	72 (49.0)	
25–33	75 (51.0)	
Race		
African American, Black	108 (73.5)	
White, Caucasian	10 (6.8)	
Native American, American Indian, Alaska Native	1 (0.7)	
More than 1 race	16 (10.9)	
Other	9 (6.1)	
Unknown	3 (2.0)	
Site		
Hahnemann	48 (32.7)	
Temple	55 (37.4)	
Einstein	44 (29.9)	
Health status (i.e., self-rated health)		
Good	103 (70.1)	
Poor	41 (27.9)	
Adverse childhood experiences (ACE)		
ACE score		4.5 (2.9)
Categories		
Low, 0–2 ACES	47 (32.0)	
Moderate, 3–5 ACEs	41 (27.9)	
High, 6–10 ACEs	57 (38.8)	
Posttraumatic stress disorder (PTSD)		
PCL-5 score		37.1 (18.8)
Provisional PTSD (PCL-5 score ≥ 33)		
Yes	93 (63.3)	
No	54 (36.7)	

Due to missing data, some percentages do not sum to 100. Study setting: Philadelphia, PA; 2014–2019

*SD* standard deviation

### Individual ACEs and Provisional PTSD Case Status

Estimated odds ratios for positive provisional PTSD case status per individual ACE are presented in the study’s supplemental material accessible online (Table A2). All ACEs, except for parental separation/divorce and domestic violence, demonstrated a significant positive relationship with provisional PTSD, though the strength of these positive associations varied. Emotional neglect demonstrated the strongest relationship with risk of provisional PTSD, with affirming participants at an increased risk of 39% (OR = 1.39; 95% CI = 1.19, 1.63). Substance abuse in the household increased the risk by 36% (OR = 1.36; 95% CI = 1.16, 1.59) for alcohol and 30% (OR = 1.30; 95% CI = 1.11, 1.53) for drugs. Emotional abuse, sexual abuse, and mental illness in the household each individually contributed to an increased risk of provisional PTSD by approximately 30%. While physical abuse resulted in an increased risk of 17%, this relationship was marginally statistically significant (OR = 1.17; 95% CI = 1.00, 1.38).

### Individual ACEs and PCL-5 PTSD Score

Estimated mean differences in PCL-5 scores per unit increase in each individual ACE (i.e., associations between individual ACEs and total PCL-5 score) are presented in the study’s supplemental material accessible online (Table A3). Emotional neglect demonstrated the strongest significant effect, correlating with increased PCL-5 scores. Following, in order of descending strength, were emotional abuse, sexual abuse, and mental illness in the household (separately); substance abuse in the household (alcohol); incarcerated household member; substance abuse in the household (drugs); and domestic violence. Each aforementioned ACE separately increased PCL-5 scores significantly. Although physical abuse and parental separation or divorce resulted in increased scores, these relationships were not statistically significant.

### Cumulative ACEs and Provisional PTSD Case Status

Results from models examining cumulative ACEs and provisional PTSD case status can be found in Table 2. Results from the first model (variables: total ACE score and positive provisional PTSD case status) demonstrated a significant positive relationship such that as total ACE scores increased, the odds of having provisional PTSD also increased. Specifically, for 1 additional increase in the total ACE score, there was a 7% increase in the odds of having provisional PTSD (OR = 1.07; 95% CI = 1.04, 1.09). With respect to the second model (variables: categories of ascending ACE exposure and positive provisional PTSD case status), there was a 27% increase in the likelihood of having provisional PTSD among the moderate ACE category versus the low ACE exposure group (OR = 1.27; 95% CI = 1.05, 1.53). For the high ACE category, that risk doubled to 54% (OR = 1.54; 95% CI = 1.28, 1.84). In both models, sex, age, site, and self-rated health were not statistically significant.

**Table 2:** Estimated associations for the odds of provisional PTSD (PCL-5 score ≥ 33)

**ACE interpretation**	**Total score (numeric)**		**Category (low-high)**	
	*OR*	*95% CI*	*OR*	*95% CI*
Intercept	1.35	(1.08, 1.69)	1.41	(1.14, 1.76)
Sex				
Female (reference)				
Male	0.89	(0.76, 1.04)	0.89	(0.76, 1.04)
Age				
25–33 (reference)				
18–22	1.04	(0.90, 1.20)	1.04	(0.90, 1.20)
Site				
Temple (reference)				
Hahnemann	1.13	(0.94, 1.36)	1.14	(0.95, 1.37)
Einstein	1.14	(0.96, 1.36)	1.12	(0.93, 1.33)
Self-rated health				
Good (reference)				
Poor	1.07	(0.91, 1.25)	1.09	(0.93, 1.28)
Adverse childhood experiences				
Total ACE score	1.07	(1.04, 1.09)	NA	NA
ACE category				
Low, 0–2 ACES				
Moderate, 3–5 ACEs	NA	NA	1.27	(1.05, 1.53)
High, 6–10 ACEs	NA	NA	1.54	(1.28, 1.84)

Statistical technique: logistic regression model. Study setting: Philadelphia, PA; 2014–2019

*PTSD* posttraumatic stress disorder, *ACE* adverse childhood experience, *OR* odds ratio, *CI* confidence interval, *NA* not applicable (i.e., not included in indicated model).

### Cumulative ACEs and PCL-5 PTSD Score

Results from models examining cumulative ACEs and PCL-5 score can be found in Table 3. There was a significant positive relationship between total PCL-5 score and total ACE score, such that, as participants’ total ACE scores increased, their PCL-5 scores increased (*b* = 0.16; *p* < 0.05). When considering the categorical measure of ACEs, those in the moderate ACE category had significantly increasing total PCL-5 scores (*b* = 0.52; *p* = 0.01). Furthermore, the estimated mean differences appeared to double for those in the high ACE category (*b* = 1.02; *p* < 0.05). Both models yielded significant positive relationships between PCL-5 scores and self-rated health, such that those in the “poor” self-rated health category had increasing scores (compared to those participants in the good self-rated health category). Males had significantly lower scores compared to females in both models considered.

**Table 3:** Estimated mean differences in PCL-5 scores per unit increase in independent variables

**ACE interpretation**	**Total score (numeric)**			**Category (low-high)**		
	*b*	*SE*	*p*	*b*	*SE*	*p*
Intercept	−0.87	0.22	0.00	−0.68	0.22	0.00
Sex						
Female (reference)						
Male	−0.33	0.15	0.03	−0.36	0.16	0.03
Age						
25–33 (reference)						
18–22	0.13	0.14	0.34	0.14	0.14	0.34
Site						
Temple (reference)						
Hahnemann	0.25	0.18	0.16	0.28	0.18	0.13
Einstein	0.31	0.17	0.07	0.25	0.18	0.15
Self-rated Health						
Good (reference)						
Poor	0.35	0.16	0.03	0.42	0.16	0.01
Adverse childhood experiences						
Total ACE score	0.16	0.03	0.00	NA	NA	NA
ACE category						
Low, 0–2 ACES						
Moderate, 3–5 ACEs	NA	NA	NA	0.52	0.19	0.01
High, 6–10 ACEs	NA	NA	NA	1.02	0.18	0.00

Statistical technique: linear regression model. Study setting: Philadelphia, PA; 2014–2019

*PTSD* posttraumatic stress disorder, *ACE* adverse childhood experience, *SE* standard error, *NA* not applicable (i.e., not included in indicated model).

## Discussion

African American youth are at higher risk of being a survivor of violence in the USA [3, 4]. Urban areas have higher rates of interpersonal violence, especially in segregated neighborhoods that have been historically oppressed [4, 6]. This study sought to understand the associations between individual and cumulative ACEs and PTSD among young adults in Philadelphia, PA. We observed that higher cumulative levels of ACEs were associated with higher levels of PTSD symptoms at baseline. This relationship held whether the outcome was defined as provisional PTSD or by total PTSD symptom scores.

We also found that medium and high levels of ACEs were more likely to be associated with provisional PTSD compared with low levels of ACEs. Individual ACEs contributed differently to PTSD symptoms at baseline with emotional neglect having the strongest association. Household substance abuse, emotional abuse, and household mental illness were also significantly associated with PTSD symptoms. Overall, these relationships held whether PTSD symptoms were measured using provisional PTSD status or the continuous PCL-5 score.

Our findings are consistent with other research which shows a relationship between childhood adversity and vulnerability for PTSD in later life [14, 15, 43–45]. Prior studies have revealed both high levels of PTSD symptoms and high ACE scores among survivors of urban violence [46, 47]. However, few studies have explored these relationships among a cohort of young survivors of violence, most of whom are young people of color. Our study makes a unique contribution by identifying that at presentation for medical treatment of violent injury, survivors with higher ACE scores also have higher PTSD symptom scores.

Individuals who are survivors of interpersonal violence and are exposed to violence are at higher risk for re-injury and trauma recidivism [48]. HVIPs recruit survivors from trauma centers and address basic, social, and psychological needs to facilitate recovery. HVIPs connect with survivors who are impacted by trauma and ACEs to provide needed social supports [48–50]. Research has found that connecting survivors of interpersonal violence to social and economic services can increase formal help-seeking [50], and can moderate the association between ACEs and health outcomes [51–53].

Given that there is evidence of HVIPs being beneficial for patients who are exposed to and who are survivors of interpersonal violence [54–57], HHP’s trauma informed approach has the potential to interrupt the cycle of violence by connecting individuals with social and economic resources [25, 57]. HVIPs can also improve the lives of those who have exposure to and are survivors of interpersonal violence [58, 59].

Although this study has a number of noted strengths and adds to the body of evidence around the importance of ACEs exacerbating the development of PTSD in young survivors of violence, this study has several limitations. First, this study was based on cross-sectional data collected at the time of presentation to the emergency department for injured youth. Because of this, we are not able to draw causal inferences regarding the relationship between early childhood adversity and PTSD symptoms. A second limitation relates to our sample size. Initial power calculations assumed a moderate effect size for an odds ratio of 1.5, with 147 study participants, using logistic regression to examine the association between PTSD and ACEs. Power was assumed to be approximately 70%, and, while we found a statistically significant association in the odds of provisional PTSD, our estimated OR for the effect of ACE (by way of total ACE score) was relatively small at 1.07 (95% CI: 1.04, 1.09). Third, because the CDC ACEs questionnaire depends on participant self-report, suppressed traumatic memories or stigmatizing experiences such as sexual victimization may be underreported.

### Public Health Implications

This study confirms that young survivors of violence are at high risk of both childhood adversity and PTSD. It is essential to provide resources to survivors of urban violence given our knowledge that trauma can exert detrimental effects on physical and psychological health. Hospital-based violence intervention programs (HVIP) have been shown to decrease symptoms of trauma. Future research on ACEs, PTSD, and HVIP should seek to understand how early intervention among survivors of violence can decrease PTSD for those who also carry the burden of childhood adversity.

## Supplementary Information

ESM 1 (DOCX 27 kb)
