# Supplementary material for: Examining Associations between Adverse Childhood Experiences and Posttraumatic Stress Disorder Symptoms among Young Survivors of Urban Violence
Source: J Urban Health. 2022 Jun 14;99(4):669–79. doi: 10.1007/s11524-022-00628-4 (PMC9360210; doi:10.1007/s11524-022-00628-4)
Supplement: Supplementary file 1 — (DOCX 27 kb) [file 11524_2022_628_MOESM1_ESM.docx]

**Supplemental Material**

Table A1. Definition and scoring of each category of adverse childhood experiences (ACE)^35,36^

| **ACE Category** | **Question** | **Affirmative** | **Negative** |
| --- | --- | --- | --- |
| Emotional abuse | How often did a parent or adult in your home ever swear at you, insult you, or put you down? | - Once - More than once | - Never - Don’t know/Not sure - Prefer not to answer |
| Physical abuse | How often did a parent or adult in your home ever hit, beat, kick, or physically hurt you in any way? Do not include spanking. | - Once - More than once | - Never - Don’t know/Not sure - Prefer not to answer |
| Sexual abuse | - How often did anyone at least 5 years older than you or an adult, ever touch you sexually? - How often did anyone at least 5 years older than you or an adult, try to make you touch them sexually? - How often did anyone at least 5 years older than you or an adult, force you to have sex? | - Once - More than once | - Never - Don’t know/Not sure - Prefer not to answer |
| Mental illness in the household | Did you live with anyone who was depressed, mentally ill, or suicidal? | Yes | - No - Don’t know/Not sure - Prefer not to answer |
| Incarcerated household member | Did you live with anyone who served time or was sentenced to serve time in a prison, jail, or other correctional facility? | Yes | - No - Don’t know/Not sure - Prefer not to answer |
| Substance abuse in the household (Alcohol) | Did you live with anyone who was a problem drinker or alcoholic? | Yes | - No - Don’t know/Not sure - Prefer not to answer |
| Substance abuse in the household (Drugs) | Did you live with anyone who used illegal street drugs or who abused prescription medications? | Yes | - No - Don’t know/Not sure - Prefer not to answer |
| Parental separation or divorce | Were your parents separated or divorced? | Yes | - No - Don’t know/Not sure - Prefer not to answer |
| Domestic violence | How often did your parents or adults in your home ever slap, hit, kick, punch or beat each other up? | - Once - More than once | - Never - Don’t know/Not sure - Prefer not to answer |
| Emotional neglect | How often did you feel that no one in your family loved you or thought you were important or special? | - Once - More than once | - Never - Don’t know/Not sure - Prefer not to answer |

Total ACE score has a range of 0 to 10. The following sub-ACE measures contribute 1 point: emotional abuse, physical abuse, mental illness in the household, incarcerated household member, parental separation or divorce, domestic violence, emotional neglect. The substance-abusing household member contributes 2 points for the two different questions. For the sexual abuse questions, if participant answered affirmative to at least one of the questions, sexual abuse ACE sub measure only contributes 1 point.

Table A2. Estimated associations (OR and 95% CI) between individual ACE and the odds of positive provisional PTSD case status (PCL-5 Score ≥ 33) each individual ACE

| **Individual ACE Measure** | **OR** | **95% CI** |
| --- | --- | --- |
| Emotional abuse | 1.30 | (1.11, 1.52) |
| Physical abuse | 1.17 | (1.00, 1.38) |
| Sexual abuse | 1.27 | (1.06, 1.51) |
| Mental illness in the household | 1.33 | (1.13, 1.56) |
| Incarcerated household member | 1.21 | (1.03, 1.42) |
| Substance abuse in the household (Alcohol) | 1.36 | (1.16, 1.59) |
| Substance abuse in the household (Drugs) | 1.30 | (1.11, 1.53) |
| Parental separation or divorce | 1.02 | (0.86, 1.21) |
| Domestic violence | 1.15 | (0.97, 1.35) |
| Emotional neglect | 1.39 | (1.19, 1.63) |

PTSD = posttraumatic stress disorder; ACE = adverse childhood experience; OR = odds ratio; CI = confidence interval. Statistical technique: Logistic regression model. Study setting: [Identifier 29; clarification: Location, per AJPH table requirements]; 2014-2019.

Table A3. Estimated mean differences (β and 95% CI) in PCL-5 scores per unit increase in each individual ACE

| **Individual ACE Measure** | **b** | **95% CI** |
| --- | --- | --- |
| Emotional abuse | 0.65 | (0.33, 0.96) |
| Physical abuse | 0.26 | (-0.07, 0.59) |
| Sexual abuse | 0.65 | (0.30, 0.99) |
| Mental illness in the household | 0.65 | (0.33, 0.97) |
| Incarcerated household member | 0.57 | (0.25, 0.88) |
| Substance abuse in the household (Alcohol) | 0.63 | (0.31, 0.95) |
| Substance abuse in the household (Drugs) | 0.54 | (0.22, 0.86) |
| Parental separation or divorce | 0.28 | (-0.06, 0.63) |
| Domestic violence | 0.51 | (0.18, 0.84) |
| Emotional neglect | 0.87 | (0.57, 1.17) |

PTSD = posttraumatic stress disorder; ACE = adverse childhood experience; CI = confidence interval. Statistical technique: Linear regression model. Study setting: [Identifier 29; clarification: Location, per AJPH table requirements]; 2014-2019.
